# An essential *Trypanosoma brucei* protein kinase: a functional analysis of regulation and the identification of inhibitors

**DOI:** 10.3389/fpara.2023.1272378

**Published:** 2023-11-14

**Authors:** Marilyn Parsons, Ben Parsons, Marissa Dean, Amy E. DeRocher, Zeba Islam, Dustin J. Maly, Bryan C. Jensen

**Affiliations:** ^1^ Center for Global Infectious Disease Research, Seattle Children’s Research Institute, Seattle, WA, United States; ^2^ Department of Pediatrics, University of Washington, Seattle, WA, United States; ^3^ Department of Global Health, University of Washington, Seattle, WA, United States; ^4^ Department of Chemistry, University of Washington, Seattle, WA, United States

**Keywords:** protein kinase, *Trypanosoma brucei*, AGC kinase, African trypanosomiasis, hesperadin, inhibitor screen, subcellular localization, complementation analysis

## Abstract

**Introduction:**

The protein serine/threonine kinase AEK1 is essential in the pathogenic stage of Trypanosoma brucei, the causative agent of African trypanosomiasis. AEK1 is a member of the AGC protein kinase family, although it is not closely related to a specific human AGC kinase. Our previous chemical genetic studies showed that targeted inhibition of AEK1 in parasites expressing analog-sensitive AEK1 blocked parasite growth and enhanced survival of infected mice.

**Methods:**

To further validate AEK1 as a drug target, we used the chemical genetic system to determine the effect of a 24 hour loss of AEK1 activity on cell viability at the clonal level. A panel of 429 protein kinase inhibitors were screened against the wild-type protein for binding, using time-resolved fluorescence energy transfer (TR-FRET). The role of phosphorylation sites and motifs was probed by determining whether expression of proteins harboring mutations in these sequences could rescue AEK1 conditional knockout parasites. To determine the effect that mutations in the phosphosites have on the kinase activity of cellular AEK1 we compared the in vitro kinase activity of mutant and wild-type proteins immunoprecipitated from parasite lysates using the exogenous substrate MBP. Finally, the tagged AEK1 protein was localized by deconvolution microscopy.

**Results:**

After a 24 hour exposure to an AEK1 inhibitory analog in the chemical genetic system, less than five percent of the remaining live cells can clonally expand, further validating AEK1 as a drug target. In the AEK1 inhibitor screening assay, we identified 17 hit compounds. Complementation studies showed that of the two known phosphorylation sites in the activation loop; mutation of one abolished function while mutation of the other had no discernable effect. Mutation of the other two AEK1 phosphosites gave intermediate phenotypes. Mutations in either the hydrophobic motif at the C-terminus of the protein or in the region of AEK1 predicted to bind the hydrophobic motif were also required for function. All parasites with defective AEK1 showed reduced proliferation and defects in cytokinesis, although the tested mutations differed in terms of the extent of cell death. Kinase activity of immunoprecipitated AEK1 phosphosite mutants largely paralleled the effects seen in complementation studies, although the mutation of the phosphosite adjacent to the hydrophobic motif had a greater impact on activity than predicted by the complementation studies. AEK1 was localized to cytoplasmic puncta distinct from glycosomes and acidocalcisomes.

**Discussion:**

The rapid loss of viability of cells inhibited for AEK1 supports the idea that a short course of treatment that target AEK1 may be sufficient for treatment of people or animals infected with T. brucei. Key regulatory elements between AEK1 and its closest mammalian homolog appear to be largely conserved despite the vast evolutionary distance between mammals and T. brucei. The presence of AEK1 in cytoplasmic puncta raises the possibility that its localization may also play a role in functional activity.

## 1 Introduction

Human African trypanosomiasis—Chagas’ disease—and the leishmaniases are neglected vector-borne diseases caused by trypanosomatid parasites. There are no approved vaccines to prevent these diseases to date. As a result, drug treatment and vector control are critical to reducing their impact. Unfortunately, the arsenal of drugs to treat and ultimately eliminate these diseases is suboptimal, limited by factors such as efficacy, resistance, and cost (Field et al., 2017). Hence, the definition and analysis of candidate drug targets remain a priority in the field. Protein kinases (PKs) are attractive targets in the development of antimicrobial agents because of the importance of many PKs in parasite biology [see, for example, Li and Wang (2006); Ma et al. (2010); Jones et al. (2014), and McDonald et al. (2018)], PK druggability, the large amount of relevant chemical matter, and the clinical success of PK inhibitors, particularly in oncology (Wu et al., 2016; Lightfoot et al., 2019). Many PK inhibitors have been characterized against hundreds of human PKs and show varying degrees of polypharmacology. Thus, known PK inhibitors provide a good starting point for testing against novel PKs. The goal for new treatments for human African trypanosomiasis is a short course of therapy, in contrast to the longer regimens for PK-targeted drugs in oncology and other chronic diseases. A short exposure should reduce harmful side effects resulting from action against host kinases.

RNA interference (RNAi) studies of the bloodstream forms of *Trypanosoma brucei* have demonstrated that the protein kinase AEK1 (Tb927.3.2440) is essential (Jones et al., 2014). We used a conditional knockout approach to genetically confirm AEK1 essentiality and chemical genetics exploiting ATP analog-sensitive alleles to chemically validate the target (Jensen et al., 2016). The replacement of methionine 138, which lies at the back of the ATP-binding site, with the small residues glycine or alanine allowed AEK1 to specifically bind large PK inhibitors (known as bumped kinase inhibitors), such as 1294. Bloodstream form *T. brucei* cells solely expressing either of the analog-sensitive alleles of AEK1 (M138G or M138A) failed to grow upon addition of 1294, ultimately dying. In these studies, when AEK1 function is abrogated, replication and segregation of the kinetoplast (the catenated mitochondrial DNA, kDNA) and nuclear DNA appear normal, but cytokinesis fails. The uncoupling of cell growth (enlargement), mitosis, and cytokinesis has been observed following genetic knockdown of several genes in the bloodstream form of *T. brucei*, for example Aurora kinase 1 (Jetton et al., 2009), variant surface glycoprotein (VSG) (Sheader et al., 2005), and the hypothetical protein Tb427.10.13790 (Mudogo et al., 2018). Chemical genetic analysis shows that 16 h after AEK1 inhibition approximately half of the parasites have aberrant DNA contents, so they are unlikely to give rise to functional daughter cells (Jensen et al., 2016). When mice infected with the M138G mutant strain were treated with 1294 for 5 days, the mice survived longer and one-third of the mice were cured (Jensen et al., 2016).

AEK1 is highly conserved across pathogenic trypanosomatids, and recent studies show that it is also essential in multiple developmental stages of *Trypanosoma cruzi* (Chiurillo et al., 2021). The catalytic domain of *T. brucei* AEK1 shows 37% sequence identity with its closest human paralog, AKT3, and the homology drops in the C-terminal extension to 19%, with stronger similarity around the short hydrophobic motif (HM) near the C-terminus. Most notably, the phosphatidylinositol-binding PH domain of AKT PKs is absent from AEK1.

The activation of the diverse AGC kinases occurs through a variety of mechanisms. Most of the human AGC kinases, including the three AKT isoforms, require phosphorylation on the PK activation loop to achieve activity (Leroux et al., 2018). Often, this is mediated by the AGC kinase phosphoinositide-dependent kinase 1 (PDK1) (Leroux et al., 2018), but some AGC kinases autophosphorylate this site in response to stimuli, or, in the case of PDK1 itself, constitutively (Pearce et al., 2010). A conserved but structurally dynamic pocket on PDK1, termed the PDK-interacting fragment (PIF) pocket, can interact in *trans* with a hydrophobic motif (HM) present on many of the AGC kinases, facilitating the PDK1-mediated phosphorylation of the activation loop of many such kinases (Pearce et al., 2010; Leroux et al., 2018). For example, the conserved leucine of the PDK1 PIF pocket interacts with the two phenylalanines located in the HM of PKA, enhancing the HM–PIF pocket interaction (Biondi et al., 2000). The HMs also interact in *cis* with their corresponding PIF pocket, which is thought to assist the phosphorylated activation loop in stabilizing the alpha C-helix in the active state (Johnson et al., 1996; Jones et al., 2014). In addition to hydrophobic residues, the HM contains an acidic residue or a serine that can be phosphorylated, the latter providing another layer of phosphoregulation. In many AGC kinases this phosphoserine is important for activity (Freeley et al., 2011). Many AGC kinases also have a phosphoregulatory site downstream of the kinase domain and just upstream of the HM (Hauge et al., 2007; Romano et al., 2009). This phosphoresidue is thought to help stabilize the interaction of the HM and PIF pocket (Biondi et al., 2000; Hauge et al., 2007). Some AGC kinases, including PKA, PKG, and PKC, also require interaction with small molecules or other proteins for activation (reviewed in Pearce (Pearce et al., 2010)).

**Figure 1:**
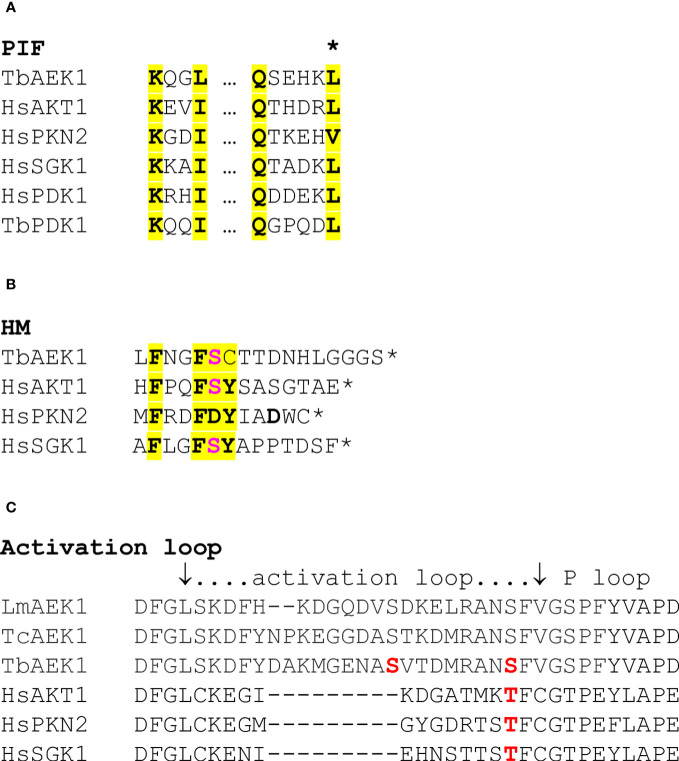
Regulatory motifs. **(A)**. The PIF pocket motif of selected AGC protein kinases. Key residues are highlighted. The two regions are separated by ≈ 32 aa. The asterisk denotes the leucine residue that intercalates between the two phenylalanines of the HM. This leucine was mutated in the studies of AEK1 described below. **(B)**. The HM. The HMs of the PKs shown in panel A are depicted. Human and *T. brucei* PDKs lack an HM. Key residues, including the phenylalanines referred to in panel A, are highlighted. The acidic/phosphorylated residues are in red bold font. The asterisk marks the end of the protein. **(C)**. The activation loop. Known phosphorylation sites are indicated in red (S221 and S229 for AEK1).

The sequence of AEK1’s N-terminal extension provides no clues to a potential regulatory function: it lacks identifiable motifs and it shows little sequence similarity across trypanosomatids (although each has a high proportion of basic residues). Trypanosomatid AEK1s contain regions related to known regulatory elements that are common to AGC kinases, including the predicted contact residues of the PIF pocket (L134) (**Figure 1A**) and the characteristic phenylalanines (F391, F394) and serine (S395) of the HM (**Figure 1B**). The analysis of the AlphaFold model for AEK1 shows that the L134 side chain interdigitates between F391 and F394 of the HM (Jumper et al., 2021; Varadi et al., 2022), which is consistent with the PKA–PDK1 interaction noted above. **Figure 1B** shows an alignment of the AEK1 HM with the HMs of several AGC kinases. Phosphoproteomic studies of *T. brucei* have identified four phosphorylation sites (Nett et al., 2009; Urbaniak et al., 2013): S221 and S229 in the activation loop (**Figure 1C**), T376 just upstream of the HM in the C-terminal extension, and S395 at the HM.

The studies reported here examine multiple aspects of AEK1 function. We show that inhibition of AEK1 prevents proliferation of apparently live cells after the inhibitor is removed, furthering its potential as a drug target. We developed a screening assay and used it to identify multiple hits in a limited screen of kinase-directed compounds. We demonstrated that regulatory mechanisms seen in many of the AGC kinases are conserved in this long-diverged protein kinase. Finally, we show that AEK1 resides in puncta of unknown origin but which could contribute to its function.

## 2 Materials and methods

### 2.1 Cell culture and transfection

All studies were performed with bloodstream forms of *T. brucei*. The single marker line, a derivative of the *T. brucei* strain 427 (Wirtz et al., 1999), and its transfectants were grown in HMI-9 with 2.5 µg/mL of G418. The parasites were transfected as described (Burkard et al., 2007), serially diluted, and selected with 2.5 µg/mL of phleomycin. The lines were considered clonal if less than one-third of the wells for a given dilution grew. Both the conditional knockout and strain expressing the M138A gatekeeper mutant protein were previously described (Jensen et al., 2016). Unless otherwise stated, all cell counts were done on a Z1 Coulter^®^ Particle Counter (Beckman Coulter) on cells diluted 1 : 1 with phosphate-buffered saline (PBS) containing 3.7% formaldehyde.

### 2.2 Washout experiments

*T. brucei* expressing only the M138A analog-sensitive AEK1 (Jensen et al., 2016) were diluted to 10^5^ cells/mL and 1294 was added to a final concentration of 1 µM, with a final concentration of 0.005% dimethyl sulfoxide (DMSO). After either 8 h or 24 h of growth in the compound or solvent control, motile cells were quantitated using a hemocytometer. The cells were diluted in a medium supplemented with 20% fetal bovine serum (FBS) and plated at 0.1 to 30 motile cells per well in 200 µL in a 96-well plate (with 48 wells per dilution). The maximum final concentration of 1294 in the diluted wells was less than 11 nM, which has no effect on the growth of the cell line. After 7 days in a 37°C CO_2_ incubator, wells were inspected and scored for presence or absence of growing cells. The percentage of wells with no growing cells was plotted versus the number motile cells plated per well. The data points where all wells, zero wells, or one well contained growing cells were eliminated from the analysis. Using Microsoft Excel^®^ (Microsoft Corporation, Redmond, WA, USA), the data points were then fitted with an exponential curve according to the equation *e* ^−^ *^mx^*, where *m* equals the plating efficiency, which reflects the proportion of the treated cells that are viable and able to clonally expand, as derived from the zero term of the Poisson distribution.

### 2.3 Plasmid construction and site-directed mutagenesis

Two separate expression plasmids were generated for the expression of the protein in *Escherichia coli*. For the first construct, *AEK1* was amplified with primers 1 and 2 (**Supplementary Table 1**) and cloned into the plasmid pET-DuetST (kindly supplied by SSGCID) such that the expressed protein contained an N-terminal 6×His-tag. Primers 3 and 4 were used to delete sequences between the end of *AEK1* and a downstream Strep-tag, allowing for the expression of a protein with an N-terminal 6×His-tag and a C-terminal Strep-tag. This allowed for the purification of the full-length protein, as approximately half of the AEK1 protein expressed in *E. coli* was truncated at the C-terminus. For the second expression plasmid that was used for the expression of protein with a C-terminal 6×His-tag, *AEK1* was amplified with primers 5 and 6 and inserted via Gibson assembly into pJX (kindly supplied by SSGCID) that had been linearized by PCR with primers 7 and 8. Site-directed mutagenesis was performed using the primers listed in **Supplementary Table 1**—primers using either QuikChange^®^ mutagenesis (Agilent) or Gibson assembly (New England Biolabs).

To assess the biological effects of specific amino acid changes, we used the plasmid pHD1344-AEK1-THP (Jensen et al., 2016), which constitutively expresses AEK1-HA following integration of the construct into the tubulin locus. Site-directed mutagenesis was performed as above.

### 2.4 Protein production

The His-tagged AEK1 proteins were expressed and purified from *E. coli* using a nickel column, as described by Bryan et al. (2011). His-AEK1-Strep was further concentrated on a MacroSep™ column (Pall) with a 10-kDa molecular weight (MW) cutoff value to approximately 5 mL and then loaded onto a Strep-Tactin^®^ XT column (IBA Lifesciences GmbH). The columns were washed five times with one column volume of SGPP (5% glycerol, 300 mM NaCl, 20 mM HEPES, pH 7.0). The AEK1 was eluted in three steps with SGPP containing 50 mM biotin. Step 1 was a 0.6 column volume, step 2 was a 1.6 column volume (containing the majority of the tagged protein), and step 3 was a 0.8 column volume.

### 2.5 Kinase inhibitor screen

In the assay, a europium-conjugated anti-6×His antibody (LANCE Eu-W1024 Anti-6×His, Perkin Elmer) is directed against the His-tag of the bacterially expressed AEK1. Europium is excited at 340 nm and emits at 615 nm. The emitted light then excites the low-affinity fluorescent probe SCP-2 [as described in Chakraborty et al. (2019)] bound at the ATP-binding site, which subsequently emits light at 655 nm to be measured. The addition of a higher-affinity test compound displaces the probe, leading to a loss of signal from fluorescent energy transfer (FRET). The compounds screened are from the SelleckChem Kinase Inhibitor Library, which mainly includes inhibitors that act at the ATP-binding site.

For the TR-FRET assay 1 µM of inhibitor (initial concentration = 25 µM in DMSO) was added to 19 µL of an assay buffer containing AEK1 (120 nM), 1.3 nM LANCE Eu-W1024-labeled anti-6×His antibody (PerkinElmer, MA, USA), 1.6 µM SCP-2, 50 mM Tris (pH 8.0), 150 mM NaCl, and 1 mg/mL of bovine serum albumin (BSA). The assays were incubated for 1 h in a 384-white assay plate (Proxiplate-384 Plus; #6008280) and spun down at 2,000 rpm for 2 min at 22°C prior to fluorescence measurement. The plate was scanned for FRET with an EnVision® Multilabel Reader (PerkinElmer, MA, USA) using a 340-nm excitation filter and emission filters at 615 nm and 665 nm. The percent reduction in FRET observed relative to the same assay buffer containing DMSO was calculated. All assays were performed in triplicate.

### 2.6 Thermal melt and thermal shift assays

For the thermal melt studies to assess protein structural integrity we used the Protein Thermal Dye Shift Kit (Applied Biosystems), following the kit instructions. The final AEK1 concentration was 2.5 µM with or without inhibitor at a 10-fold molar excess. The final DMSO concentration was 10%. The plates were read on a StepOne Real-Time PCR system (Applied Biosystems). For the data shown in Section 3.2, AEK1 had a C-terminal 6×His-tag, whereas the experiments shown in Section 3.3 used protein with both an N-terminal 6×His-tag and a C-terminal Strep-tag.

### 2.7 Western blotting

Parasite lysates were generated, and proteins (5 × 10^6^ cell equivalents) were resolved by SDS-PAGE and transferred to nitrocellulose as described (Jensen et al., 2003). V5-tagged proteins were detected with the mouse monoclonal anti-V5 antibody clone SV5-Pk1 (Thermo Fisher Scientific) at 0.3 µg/mL. The hemagglutinin (HA)-tagged proteins were detected with either mouse clone 16B12 (BioLegend) or rat clone 3F10 (Roche) monoclonal anti-HA antibodies at 1 µg/mL or 12.5 ng/mL, respectively. Rabbit polyclonal antibodies to either phosphoglycerate kinase (PGK) (Parker et al., 1995) or CK2α (Jensen et al., 2007) were used at a ratio of 1 : 10,000 and 1 : 5,000, respectively. The bound antibodies were revealed with either IRDye 680 dye-conjugated goat anti-rabbit Ig (Li-COR) or IRDye 800CW dye-conjugated goat anti-mouse Ig (Li-COR) at 25 ng/mL, and data were visualized on a Li-COR Odyssey^®^.

### 2.8 Microscopy

#### 2.8.1 Immunofluorescence assays

The parasites expressing tetracycline(Tet)-induced AEK1(M138A)-V5 [*aek1* cKO/*AEK1*(M138A-HA)] were grown overnight with or without 1 µg/mL of Tet. Cells were pelleted and washed with PBS containing 10 mM glucose before being pelleted. The cell pellets were then resuspended in PBS with 10 mM glucose at 5 × 10^8^ cells/mL and 40 µL was spotted onto poly-L-lysine-coated coverslips. The coverslips were incubated for 30 min at room temperature to allow cells to bind. Subsequently, 500 µL of ice-cold methanol was added to the coverslips and the cells were incubated for an additional 30 min. The incubation and washing steps for antibody staining were as described in Jensen et al. (2021). The antibodies used were mouse anti-V5 at 5.5 μg/mL, the glycosomal marker rabbit anti-PGK at 1 : 300, and the acidocalcisome marker rabbit anti-vacuolar pyrophosphatase at 1 : 400 (kindly gifted by Roberto Docampo). The secondary antibodies were anti-mouse IgG2-fluorescein isothiocyanate (FITC) (Southern Biotechnology) and anti-rabbit Ig Texas red (Southern Biotechnology). After incubations and washing, slides were mounted using Prolong glass with NucBlue™ (Thermo Fisher Scientific). The cells were viewed on a DeltaVision Elite microscope with an Olympus PlanApo N 60×/1.42 oil PSF ∞/0.17/FN 26.5 objective. The image stacks were deconvolved using GE Healthcare’s softWoRx 7.0 10 cycles ratio method. The multiple images of the negative control samples were analyzed to measure the background fluorescence intensity for anti-V5 staining and the resulting value was used as the lower limit of intensity for scaling of the AEK1-V5 signal.

#### 2.8.2 Viability and morphologic analysis

Two microscopic methods were used to assess cell viability: dye exclusion for the growth analysis and cell motility for the washout experiments. For the growth analysis, parasites were concentrated by centrifugation at 5,000 × g for 10 min. The parasites (unfixed) were incubated with 10 µM ethidium homodimer for 10 min and spotted onto slides. They were visualized on a fluorescence microscope with at least 100 cells being scored as alive (non-fluorescent) or dead (stained). For the washout analysis, live cells (defined as motile) were quantitated using a hemocytometer.

The parasites were also scored for morphology, categorized as being trypanosome-like (being relatively long and having a pointed anterior), monsters (rounded and typically multiflagellated), or dead/debris. Both trypanosome-like and monster cells exclude ethidium homodimer.

#### 2.8.3 Cell cycle analysis

The parasites were pelleted and resuspended in PBS with 4% formaldehyde. The cells were immediately spotted onto poly-L-lysine-coated slides. After 60 min at room temperature, the unbound cells were aspirated, and cells were permeabilized by adding PBS with 0.1% Triton X-100. Following a 10-min incubation, slides were washed two times with PBS before staining with 4′,6-diamidino-2-phenylindole (DAPI) at 10 µg/mL in PBS for 30 min. The slides were washed two times with PBS and mounted with ProLong™ Gold Antifade Mountant (Thermo Fisher Scientific). For each line at least 200 cells were visualized by fluorescence microscopy and characterized for the number of nuclei and kinetoplasts. In a small fraction of multinucleate cells (< 10%) it was difficult to determine exactly how many kinetoplasts were present.

### 2.9 Immunoprecipitation and kinase assays

The protein lysates from the bloodstream forms of the parasites were prepared, as described by Das et al. (1998), except that the stated proteases and phosphatase inhibitors were replaced with cOmplete™ Mini EDTA-free protease inhibitor (Roche) and PhosSTOP (Roche). The HA-tagged AEK1 from 1.8 × 10^8^ cells were immunoprecipitated, as described by Park et al. (2002), using 2 µg of mouse anti-HA clone16B12 (BioLegend) and 40 µL of sheep anti-mouse Dynabeads (Invitrogen). For the kinase assays, the beads were washed once in kinase buffer (40 mM Tris pH 7.5, 20 mM MgCl_2_, and 0.1mg/mL of BSA). The kinase assays were performed, as described by Park et al. (2002), using ^32^P-γ-ATP and dephosphorylated myelin basic protein (MBP, MilliporeSigma), except that the kinase buffer was replaced with the same buffer described above. One-third of each reaction was resolved by SDS-PAGE. The gels were stained with Coomassie, fixed, and dried, and then labeled proteins were detected by phosphorimaging. The signals were quantified using ImageJ (Schneider et al., 2012). To normalize the amount of immunoprecipitated HA-tagged protein, one-quarter of each reaction was analyzed by Western blot analysis, with blots probed with rat anti-HA antibodies.

## 3 Results

### 3.1 Washout assays demonstrate irreversible damage by AEK1 inhibition

As noted above, about half of the parasites expressing analog-sensitive AEK1(M138A) show aberrant DNA content after treatment with 1294 for 16 h and have probably lost their ability to proliferate. Thus, a simple assay determining the number of live cells would overestimate the percentage of cells that are able to proliferate following inhibition of AEK1 activity. To determine the number of cells that have retained the ability to proliferate, we used a limiting dilution analysis, choosing a drug concentration and exposure time that preliminary experiments indicated would reduce viability to approximately 20% of the solvent control. Parasites solely expressing AEK1(M138A) (Jensen et al., 2016) were treated with 1 µM 1294 or vehicle control for 24 h (except as noted). For each condition, the total number of motile cells was determined using a hemocytometer. As seen in **Table 1**, two or three doublings took place in the control cultures, and almost all motile cells were able to undergo clonal expansion. After a 24-h treatment with 1294, the number of motile cells solely expressing AEK1(M138A) increased about 50% from the beginning of the experiment, showing that some cell division had occurred in the treated cells. However, following treatment with 1294, on average, only 25% of the cell-like particles were motile. Of these, only 4.5% were able to form clones. Altogether, the total number of clones after a 24-h exposure to 1294 dropped by over 99% compared with the control. The single experiment with an 8-h drug treatment yielded a relatively mild growth defect and an 80% decrease in total clone number.

**Table 1:** Viability and proliferative potential following AEK1 inhibition.

	Treatment	Duration (h)	Fold increase, total cells	Live	Fold increase, live cells (% control)	Plating efficiency of live cells	Total clones/mL
Exp 1	DMSO	24	6.7	74%	4.9 (100%)	107%	5.2 × 10^5^
	1294	16H DMSO+ 8 h 1294	4.4	63%	2.8 (56%)	35%	1.7 × 10^5^
	1294	24	1.6	11%	0.2 (3.6%)	3.3%	5.9 × 10^2^
Exp 2	DMSO	24	9.9	94%	9.3 (100%)	107%	9.9 × 10^5^
	1294	24	1.6	16%	0.3 (2.8%)	3.5%	9.1 × 10^2^
Exp 3	DMSO	24	4.8	99%	4.8 (100)	106	5 × 10^5^
	1294	24	1.4	43%	0.1 (2.9%)	10.5%	1.44 × 10^4^

a The starting cell concentration was 10^5^/mL.

### 3.2 Screening of a kinase-directed compound library

While bumped kinase inhibitors such as 1294 are useful tools for analyzing function using analog-sensitive mutant kinases, they are not inhibitors of wild-type (WT) AEK1 and hence are not suitable leads for AEK1 drug discovery. We therefore attempted to develop an activity-based assay amenable to high-throughput screening. We were able to express and purify full-length protein in *E. coli* (see Materials and methods). Unfortunately, in assays suitable for medium throughput screening, this protein and also the same protein bearing various phosphomimetic substitutions showed little to no activity toward exogenously added substrates and negligible autophosphorylation. The functional studies described below suggest that this is probably due at least in part to the requirement of phosphorylation of serine 229, which cannot be mimicked by a phosphomimetic substitution. We attempted to create AEK1 bearing a phosphoserine at residue 229 using an amber suppressor tRNA specific for phosphoserine (Park et al., 2011; Balasuriya et al., 2018), but were unable to generate significant levels of full-length protein.

In lieu of an activity-based assay, we used a Lantha TR-FRET-based assay to screen for compounds that displaced a probe bound to the ATP-binding site of AEK1. We assayed 429 compounds from the Selleckchem Kinase Inhibitor Library in triplicate at 1 µM (eightfold molar excess over AEK1) and identified 17 compounds that reduced the signal by at least 50% (**Table 2** and **Supplementary Table 1**). Interestingly, the top hit, hesperadin, is a known inhibitor of AUK1, the *T. brucei* homolog of Aurora kinase (Jetton et al., 2009).

**Table 2:** Results and confirmation of compound screen.

Compound	% control^a^	SEM	Thermal shift (°C)^b^	Primary mammalian target(s)^c^
Hesperadin	2.6	3.0	11.0 ± 0.2	Aurora B
R406 (free base)	12.6	3.5		Syk
R406	14.6	4.6	7.6 ± 0.5	Syk
TAK-901	20.1	4.7		Aurora A/B
PF-477736	23.1	3.8		Chk1
Cediranib (AZD2171)	36.6	4.4		VEGFR(KDR)
PF-3758309	38.2	5.5		PAK4
AZD1480	43.5	9.5	6.9 ± 0.3	JAK2
SGI-7079	43.7	1.2		Axl
AZ 960	43.7	4.2		JAK2
Foretinib (GSK1363089)	44.0	6.1		HGFR, VEGFR
AMG-900	44.3	6.3		Aurora A/B/C
PF-431396	44.9	2.5		PYK2,FAK
BX-912	47.1	6.7	6.5 ± 0.3	PDK1
Axitinib	47.6	10.2		VEGFR1/2/3, PDGFRβ, c-Kit
AT9283	48.0	8.0		JAK2/3
Danusertib	75.8	3.5	5.2 ± 0.3	Aurora A/B/C
Y-27632	97.3	3.9	1.0, 1.3	ROCK1/2
BIO	97.8	6.3	0.3, 0.5	GSK-3
Bosutinib	99.1	7.5	−0.5, −0.2	Src/Abl

a The reduction in the FRET signal with the test compound compared with the solvent control.

b The reduction in fluorescence with the compound vs. the solvent control. The T_m_ of AEK1 in the solvent was 48.6°C.

^c^Derived from www.selleckchem.com and references therein.

To confirm binding to AEK1, we tested five of the compounds that showed binding in the screen using differential scanning fluorimetry, also known as protein thermal shift, which detects the increased thermal stability of a protein afforded by ligand binding. In these assays, thermal melting (unfolding) is measured using a dye that fluoresces on binding to unfolded proteins. Four compounds were selected that represent different chemical scaffolds and target different PKs in mammalian cells. The fifth compound, danusertib, was chosen since, like hesperadin (the most potent hit), it is an AUK1 inhibitor (Ochiana et al., 2013), albeit one that showed only modest binding in our screen. For comparison, we also chose three compounds (Y-27632, BIO, and bosutinib) that were inactive in the Lantha screen. An example of the shift in *T*_m_ on compound binding is shown in **Figure S1**. The shifts for hesperadin and danusertib, with a greater shift seen for hesperadin, are shown. The data for all the compounds are shown in **Table 2**. The four compounds tested that were active in the Lantha screen (hesperadin, R406, AZD1480, and BX-912) all showed a shift of at least 6°C. In contrast, three compounds that were inactive in the Lantha screen showed a shift of 1°C or less. While binding between AEK1 and danusertib was detected, the shift was smaller than that of any of the more active compounds we tested. Overall, the potency of individual compounds in the protein thermal shift assay generally paralleled the potency in the TR-FRET assay, thereby confirming the initial hits.

The parasites expressing WT AEK1 are highly resistant to 1294, while paired lines expressing the analog sensitive AEK1(M138A) succumb rapidly to its toxic effects ((Jensen et al., 2016) and **Table 1**). These data provided us with an opportunity to gain some functional context for the thermal shift data. In thermal shift assays, we observed that 1294 increased the *T*_m_ of WT AEK1 by 4.1°C°C, while it increased the T_m_ of AEK1(M138A) by 14.1°C. These findings suggest that compounds with smaller *T*_m_ shifts (such as AZD1480 and BX-912) would have only modest on-target effects on AEK1 *in vivo*.

### 3.3 Evaluation of candidate regulatory sites on AEK1

#### 3.3.1 Workflow for analysis of candidate regulatory sites

**Figure 2:**
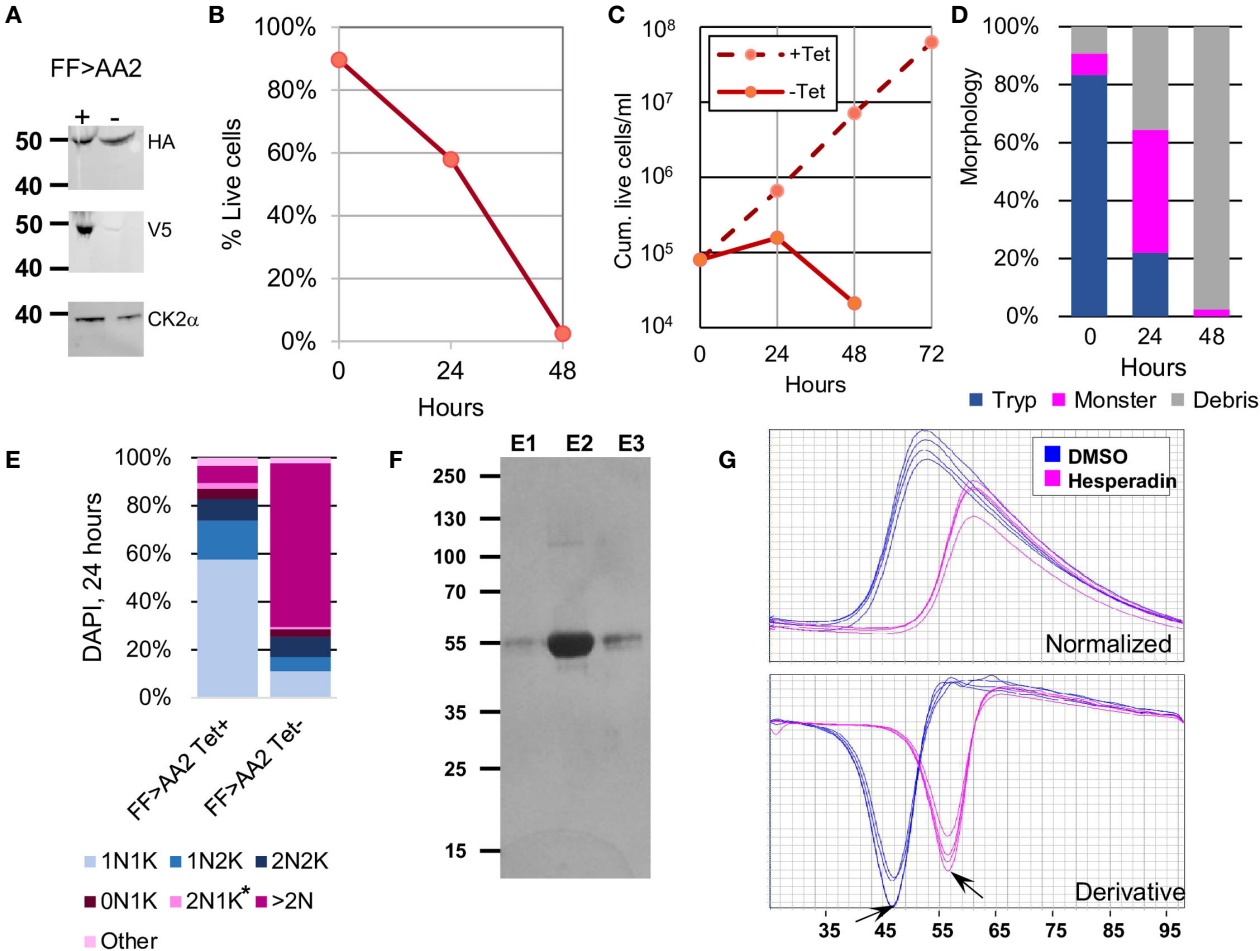
Workflow for mutant rescue analysis. The example depicts the HM mutant AEK1(F391A,F394A) clone2. **(A)**. Protein expression ± Tet. The Western blots show Tet regulation of the WT gene and expression of the test mutant. The blots were probed with an antibody to CK2α to confirm equal loading of the lanes. **(B)**. Viability in the absence of Tet over time as measured by exclusion of ethidium homodimer. Equal aliquots of triplicate samples from the growth curve shown in C were pooled for the analysis. **(C)**. Cell proliferation, as measured by cumulative live cell number, in the presence of Tet (both the WT and test protein are expressed) or the absence of Tet (only the test protein is expressed). Data represent the average of triplicate samples. The particle counts were corrected for viability as in panel B to yield the live cell count. The error bars denote one standard deviation between the cell counts. **(D)**. Morphology during Tet withdrawal. “Tryp” refers to longer cells with pointed anterior ends. Monsters are large, rounded cells with two or more protruding flagella. Prior studies have shown that these are multinucleate cells. The debris consisted of cells that were phase dark, having lost membrane integrity. **(E)**. Cell cycle analysis. The number of nuclei (N) and kinetoplasts (K) per cells as revealed by DAPI staining in cultures grown with Tet or 24 h after Tet withdrawal. The blue segments are those that make up the normal cell cycle: 1N1K, 1N2K (the kinetoplast divides before the nucleus), and 2N2K. The pink segments are abnormal forms, including those marked by an asterisk, which have a normal number of nuclei but too few or too many kinetoplasts. **(F)**. Purification of AEK1(F391A,F394A) expressed in *E. coli*. Each eluted fraction (0.5%) from the StrepTactin column was resolved by SDS-PAGE and stained with Coomassie. **(G)**. Protein thermal shift of purified AKE1(F391A,F394A) protein. The top panel is the fluorescent signal for the protein as temperature is increased. The bottom panel is the first derivative of the curve. The arrows are pointing at the peak of the derivative curve used to determine the *T*_m_ of the protein with and without hesperadin.

Given the importance of phosphorylation and the PIF–HM interactions in the function of many AGC kinases, we set out to determine whether or not these functions were also conserved in this PK from an early-diverging eukaryote. To do so, we tested whether mutant AEK1s could rescue function in the previously described conditional knockout *T. brucei* strain (Jensen et al., 2016). In this strain the two endogenous *AEK1* alleles are disrupted and the WT *AEK1* C-terminally tagged with V5 epitopes is integrated into the rDNA spacer region, where it is expressed only in the presence of Tet. The test alleles, C-terminally tagged with HA, are constitutively expressed from the tubulin locus. Thus, the withdrawal of Tet (restrictive condition) leads to the loss of the WT protein, allowing phenotypic analysis of mutated forms of AEK1. All the studies were performed with the bloodstream form of the parasites, which are the mammalian pathogenic stage. The cell cycle of the bloodstream form of *T. brucei* in our culture conditions is approximately 8 h, as was seen for the clonal lines expressing the WT AEK1 (**Figure 2A**, Tet+ condition).

**Figure 2** illustrates the workflow for functional analysis of mutations through the example of a clonal line in which the test allele contains a replacement of the two phenylalanines in the HM with alanine. At least two independent *T. brucei* clones for each mutant construct were identified that expressed the test protein. Their phenotypes were then assessed upon Tet withdrawal. Western blot analysis verified the expression of the tagged mutant protein and the appropriate Tet regulation of the WT protein (**Figure 2A**). The latter ruled out the trivial explanation that mutants that showed no phenotype survived because the WT allele had escaped Tet regulation. The growth and viability analysis, and also the morphologic characterization, were performed for all clonal lines (**Figures 2B–D**). We assessed the percentage of particles representing live cells via microscopic analysis by cellular exclusion of the ethidium homodimer. For each mutant allele that showed a growth phenotype in the rescue experiments, we quantified cell cycle phenotypes via DAPI staining for one clonal line (**Figure 2E**). During a productive cell cycle, the normal phenotypes seen are one nucleus, one kinetoplast (1N1K), 1N2K, and 2N2K. To assess overall protein folding of the mutant proteins, we expressed and purified tagged proteins in *E. coli* and then used them in thermal melt studies with and without the inhibitor hesperadin (**Figure S2** and **Table 3**). With the possible exception of the K90M kinase-dead protein, none of the mutant proteins studied showed a major change in *T*_m_ compared with the WT protein, nor did they exhibit a decreased shift on ligand binding, indicating that mutant proteins were all able to fold with no gross abnormalities.

**Table 3:** Thermal melt studies.

Protein	Growth rescue	T_m_	T_m_ + hesperadin	ΔT_m_
WT	Yes	47.8	57.4	9.6
K90M	No	45.8	53.3	7.4
L134E	No	46.5	57.2	10.7
S229A	No	47.6	60.2	12.6
T376A	Partial	49.1	62.0	12.9
F391A, F394A	No	48.1	57.6	9.5
S395A	Partial	47.9	57.5	9.6
S395D	Yes	47.7	56.8	9.1

#### 3.3.2 The hydrophobic motif and the PIF pocket

**Figure 3:**
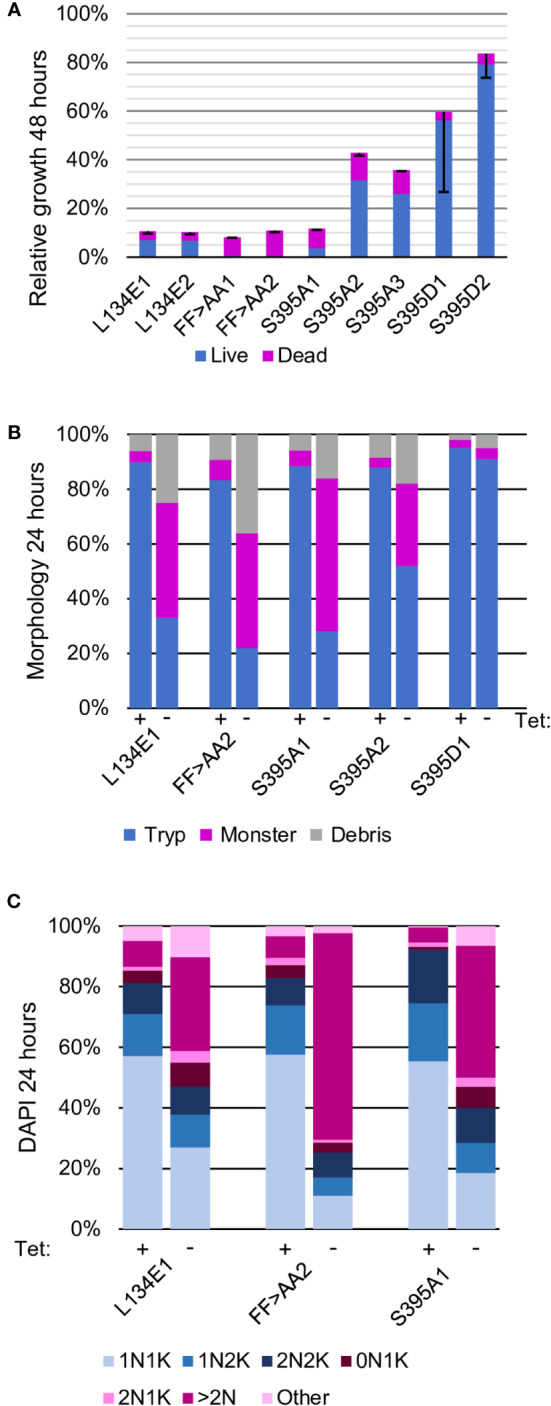
The PIF pocket and HM are essential for AEK1 function. The clones tested examined the PIF pocket (L134E), the HM phenylalanines at residues 391 and 394 (FF>AA), and the HM phosphoserine (S395A and S395D). **(A)**. Proliferation. Two independent clones for each mutant construct are depicted (three for S395A). The average percent cumulative particle counts from the cultures grown without Tet compared with the cultures grown with Tet, parsed into live (blue) and dead (pink) cell counts, are also shown. The black bars mark the standard deviation. **(B)**. Morphologic analysis. One cell line for each construct was selected for further study (two for S395A) and morphology assessed at time 0 (Tet+) and 24 h after Tet withdrawal (Tet–), as described in **Figure 2**. **(C)**. Cell cycle analysis. One cell line for each construct was scored for the number of nuclei (N) and kinetoplasts (K) per cell after growing in the presence of Tet (time 0) or 24 h after Tet withdrawal (Tet-). The blue segments represent normal cell cycle stages, and the pink segments represent abnormal forms, as described in **Figure 2**

To test whether or not the HM is required for AEK1 function, both phenylalanines (F391 and F394) were mutated to alanine. The parasites expressing AEK1(F391A,F394A) died rapidly on Tet withdrawal, with ≈ 63% of the cell-like structures in cultures being viable at 24 h and only 4% being viable at 48 h (**Figure 3A**). The morphological phenotype of the parasites paralleled what we observed for the conditional knockout (Jensen et al., 2016), with large numbers of aberrantly large parasites (“monsters”) within 1 day after Tet withdrawal (**Figure 3B**) and large amounts of debris by 2 days (data not shown). At 24 h, the DAPI staining showed that the phenotypes of the parasites in the restrictive condition (–Tet) were the same as previously observed in the *AEK1* conditional knockout—a large decrease in the percentage of cells with a single nucleus (which normally represent > 60% of the population) and many abnormally large cells with more than two nuclei and increased numbers of kinetoplasts (i.e., “monsters”). Anucleate cell-like structures with a kinetoplast (zoids) were relatively rare (**Figure 3C**). The parasites expressing only AEK1(F391A,F394A) are at least as compromised as cells lacking AEK1 (conditional knockout), suggesting that the mutant protein is completely non-functional (Jensen et al., 2016). In thermal melt studies, the *T*_m_ of AEK1(F391A,F394A) was essentially the same as the WT protein, both with and without inhibitor, indicating no gross disruption in protein folding. Taken together, these data indicate that the hydrophobic motif is essential for AEK1 function.

Similar experiments were conducted with parasites expressing AEK1 in which S395 in the hydrophobic motif was mutated to alanine (S395A), which cannot be phosphorylated. The two initial clones varied in the degree of impairment, with the first showing a stronger growth defect, more cell death, and more aberrant cells than the second clone. A third clonal line was therefore analyzed and found to have the milder phenotype. This difference is not due to differences in expression between the clonal lines as shown by Western blot analysis (**Figures S3, S4**). The clones showed slowed growth, multinucleate cells, and death, but the proportion of cells alive at 48 h was much higher than that observed for the FF > AA mutants (**Figure 3A**). In contrast, parasites expressing AEK1 with a phosphomimetic substitution of aspartic acid (S395D) showed no growth defect. Together these data support the contention that phosphorylation of S395 is important to AEK1 function *in vivo*.

We next tested whether or not the PIF pocket region, which the HM docks to in *cis* in other AGC kinases, is required for AEK1 function. Here, we mutated leucine 134 to glutamic acid, as this substitution was previously used to examine the PIF pocket of PDK1 (Biondi et al., 2000; Biondi et al., 2001). We created clonal parasite lines that constitutively express AEK1(L134E) and tested whether the mutant protein could functionally complement loss of WT AEK1 on Tet withdrawal. A strong growth phenotype was seen, and also a dramatic loss of viability, and an aberrant morphology with multinucleate cells (**Figures 3A–C**). While the growth defect of AEK1(L134E) was severe, the parasites were not as compromised as AEK1(F391A,F394A) cells. No defect in AEK1(L134E) was seen in the thermal melt studies. These data point to conservation of the PIF pocket function in AEK1 and its requirement for activity.

#### 3.3.3 The activation loop

**Figure 4:**
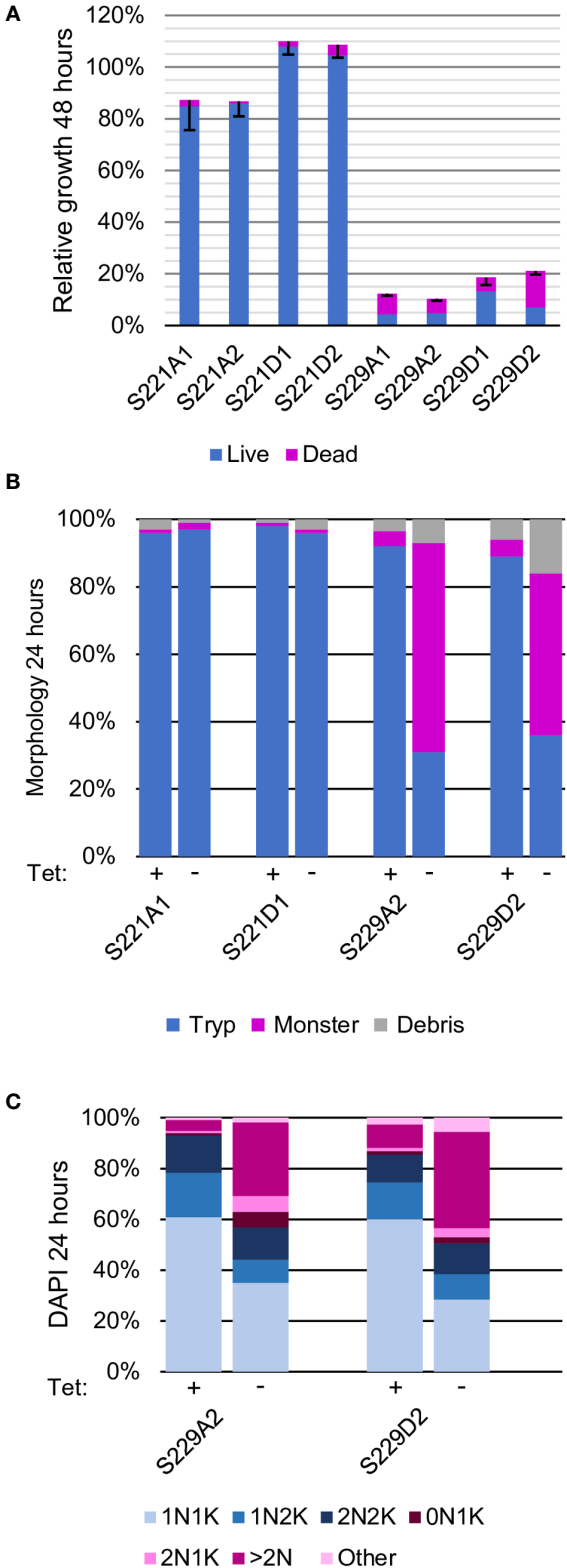
Phosphorylation of S229, but not S221, in the activation loop is essential for AEK1 function The two residues in the activation loop that are phosphorylated (S221 and S229) were tested with both alanine and aspartic (phosphomimetic) substitutions. **(A)**. Proliferation. Two independent clones for each mutant construct are depicted. The average percent cumulative particle counts from cultures grown without Tet compared with cultures grown with Tet, parsed into live (blue) and dead (pink) cell counts, are also shown. The black bars mark the standard deviation. **(B)**. Morphologic analysis. One cell line for each construct was selected for further study and morphology assessed at time 0 (Tet+) and 24 h after Tet withdrawal (Tet–), as described in **Figure 2**. **(C)**. Cell cycle analysis. One cell line for each construct was scored for the number of nuclei (N) and kinetoplasts (K) per cell after growing in the presence of Tet (time 0) or 24 h after Tet withdrawal (Tet–). The blue segments represent normal cell cycle stages, while pink segments represent abnormal forms, as described in **Figure 2**.

The next sites that were tested were the phosphorylation sites in the activation loop: S221 and S229. Serine 229 aligns with the activation loop phosphothreonine in AKT1 and AKT3, AEK1’s closest relatives in the human kinome. We generated separate site-directed serine to alanine mutations at residues S221 and S229 and expressed the corresponding proteins in the *AEK1* conditional knockout. The strains expressing AEK1(S221A) grew well after Tet withdrawal, whereas those expressing AEK1(S229A) died rapidly. In both cases, Western blots showed that Tet regulation of the WT copy was maintained and that the mutant copy was expressed (**Figures 4A–C**, **Figure S3**). Taken together, these data show that phosphorylation of S221 is not important for AEK1 function in the bloodstream forms and indicate that phosphorylation of S229 is essential. We next generated parasites separately expressing the phosphomimetic substitutions S221D and S229D. Those expressing AEK1(S221D) grew as well as the WT. In contrast, parasites expressing AEK1(S229D) died on Tet withdrawal (**Figure 4A**). Thus, the phosphomimetic aspartic acid at residue S229 was unable to substitute for the naturally occurring phosphoserine.

#### 3.3.4 The phosphorylation site in the C-terminal tail

**Figure 5:**
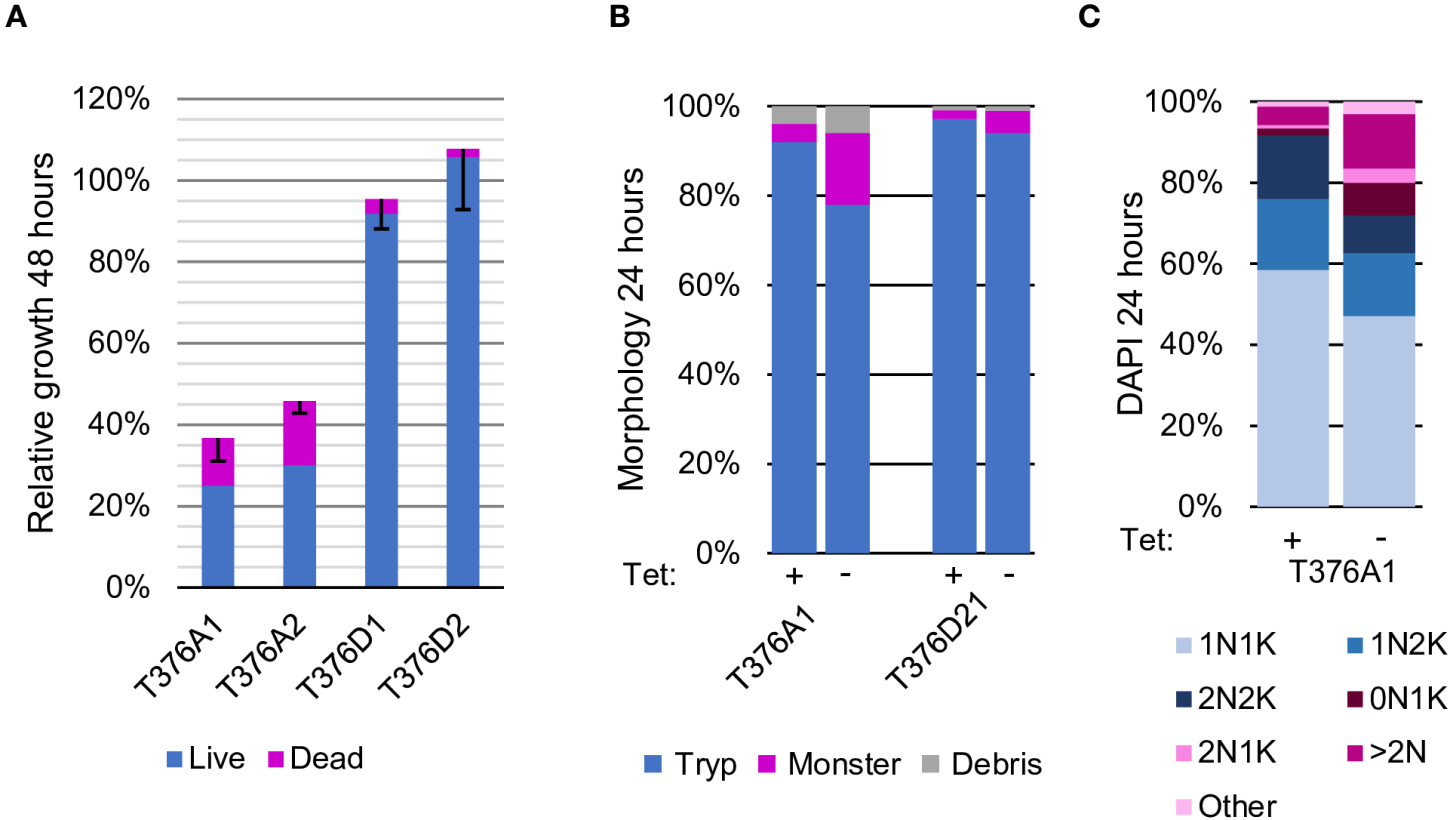
Phosphorylation of threonine in the tail region is required for normal growth and proliferation Threonine 376 was tested with both alanine and aspartic (phosphomimetic) substitutions. **(A)**. Proliferation. Two independent clones for each mutant construct are depicted. The average percent cumulative particle counts from cultures grown without Tet compared with cultures grown with Tet, parsed into live (blue) and dead (pink) cell counts, are also shown. The black bars mark the standard deviation. **(B)**. Morphologic analysis. One cell line for each construct was selected for further study and morphology assessed at time 0 (Tet+) and 24 h after Tet withdrawal (Tet–), as described in **Figure 2**. **(C)**. Cell cycle analysis. One cell line for each construct was scored for the number of nuclei (N) and kinetoplasts (K) per cell after growing in the presence of Tet (time 0) or 24 h after Tet withdrawal (Tet-). The blue segments represent normal cell cycle stages, and pink segments represent abnormal forms, as described in **Figure 2**.

In AEK1, T376, which is just upstream of the HM, is reported to be a phosphorylation site (Urbaniak et al., 2013). The parasites expressing only AEK1(T376A) continued to proliferate, but the proliferation was slowed, and abnormal forms were present, including large numbers of multinucleate cells. However, the cells were not as large nor did they have as many nuclei as those seen in rescue studies using S229A, F391A-F394A, or S395A mutants (see **Figure 5**). The expression of the WT allele was still repressed by Tet withdrawal, as shown by Western blot analysis, demonstrating that the residual proliferation can be attributed to AEK1(T376A) (**Figure S3**). The parasites expressing AEK1(T376D) grew well. Thus, it appears that phosphorylation of T376 is important for optimal AEK1 function.

### 3.4 Kinase activity of AEK1 mutants

**Figure 6:**
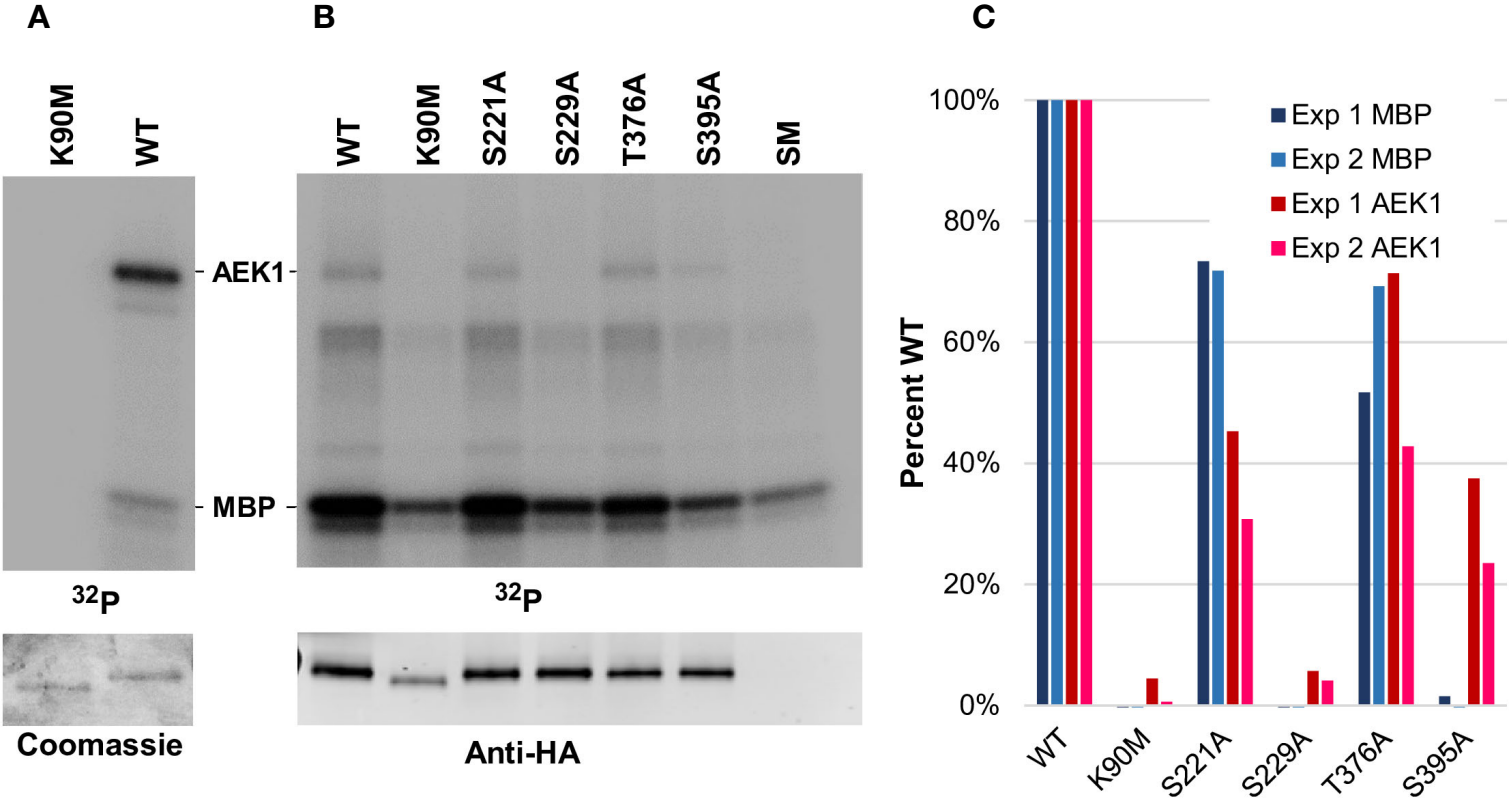
Mutant AEK1 proteins isolated from the parasites show defective enzymatic activity. **(A)**. Assay of *E. coli*-expressed AEK1. WT AEK1 and kinase-dead AEK1(K90M) were expressed and purified from *E. coli*. The radioactive assay included 1 µg of AEK1, MBP as a substrate, and γ^32^P-ATP. Top, phosphor image of the SDS-PAGE gel (one-quarter of each reaction); bottom, corresponding Coomassie-stained gel, one-quarter of each reaction. **(B)**. Assay of *in vivo*-expressed AEK1-HA. The indicated HA-tagged AEK1 proteins were immunoprecipitated from protein lysates prepared from 1.8 × 10^8^ cells expressed in the bloodstream forms of *T. brucei*. The immunoprecipitation from the parental strain single marker (SM) was used to control for background labeling. Top, phosphor image of gel. Each lane contains one-third of the reaction. Bottom, Western blot of one-quarter of each reaction probed with rat-anti-HA. **(C)**. Quantification of radiolabeling. Shown is autophosphorylation (AEK1) or transphosphorylation of the exogenous substrate (MBP), expressed as percent labeling in comparison with the WT protein. As a background control for labeling of MBP, the signal from the SM parental line was subtracted from the signal of the other lanes. Quantification of the Western blot data was used to normalize the amount of AEK1 protein immunoprecipitated.

Kinase activity assays of the WT protein isolated from *E. coli* showed some autophosphorylation activity, but only very weak phosphorylation of the exogenous substrate myelin basic protein (MBP) (**Figure 6A**). Similar results were obtained with AEK1 bearing multiple phosphomimetic residues AEK1(S229D, T376D, S395D) and AEK1(S395D) (data not shown). The lack of activity of the recombinant WT protein toward exogenous substrates most likely reflects a required phosphorylation of one or more sites, with phosphorylation of S229 being the most likely. AEK1(K90M), which has a substitution of the catalytic lysine residue in the kinase pocket, showed no autophosphorylation or other activity in the assay.

As we were not able to obtain active protein in *E. coli*, we assayed the HA epitope-tagged mutant proteins expressed in *T. brucei* and purified them by immunoprecipitation. The lysates were prepared with phosphatase inhibitors to preserve any activating phosphorylation that occurred *in vivo*. In contrast to the *E. coli*-expressed protein, the WT AEK1-HA, immunoprecipitated from parasites, showed both autophosphorylation and robust transphosphorylation activity toward the non-physiological substrate MBP (**Figures 6B, C**.) No *trans* activity toward MBP or autophosphorylation was detected in the assay of AEK1(S229A), which is consistent with its inability to rescue loss of the WT protein. Intermediate transphosphorylation activity was seen for AEK1(S221A) which fully rescues and for AEK1(T376A) which shows a modest growth phenotype. Surprisingly, although AEK1(S395A) partially rescued the loss of WT-AEK1 *in vivo* and the purified protein showed easily detected autophosphorylation, little to no MBP transphosphorylation activity was seen. No activity above the background was seen in immunoprecipitated AEK1(K90M), which lacks the active site lysine. We should note that substitutions at phosphorylation sites may disrupt phosphoregulatory circuits *in vivo*, yielding secondary effects on other phosphosites.

### 3.5 Subcellular localization of AEK1

**Figure 7:**
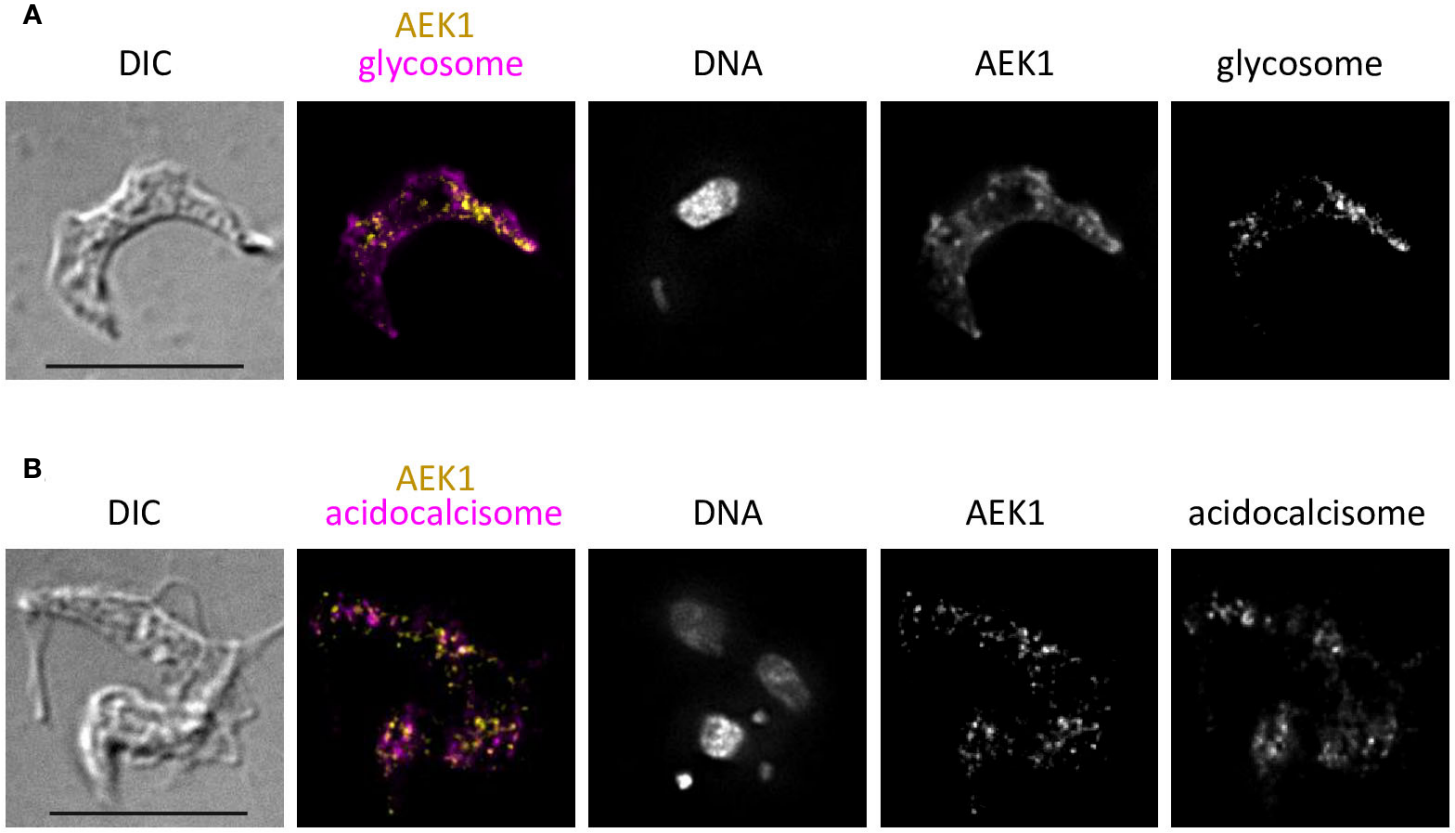
AEK1 does not co-localize with glycosomes or acidocalcisomes. The bloodstream forms of the parasites expressing AEK1(M138A)-V5 were grown overnight in the presence of Tet to induce expression of the tagged AEK1. The cells were fixed and probed with mouse anti-V5 and rabbit-test antibodies detecting organellar markers, followed by anti-mouse Ig FITC (yellow in overlay) and anti-rabbit Ig Texas red (magenta in overlay). Individual channels are shown in black and white. The pixels where yellow and magenta colocalize appear red. The samples were mounted using ProLong glass with NucBlue. DIC and fluorescence overlay and also single-channel images are presented. The deconvolution is described in Materials and methods. Bar = 10 μM. **(A)**. Cells were probed with anti-V5 and rabbit anti-PGK (glycosome marker). **(B)**. Cells were probed with anti-V5 and rabbit anti-vacuolar pyrophosphatase (acidocalcisome marker). The image is from two overlapping cells, one with two nuclei and two kinetoplasts.

The defective cytokinesis phenotype of *AEK1* knockdowns raised the possibility that the kinase could be localized to a distinct compartment of the cell. Immunofluorescence analysis of the C-terminally tagged AEK1 using DeltaVision deconvolution microscopy revealed that the protein was localized to small puncta that were distinct from glycosomes and acidocalcisomes (**Figure 7**). The protein was not present in the nucleus, nor did it align with the flagellum or associated structures. We note that the signal was relatively faint, so not all parasites showed obvious staining. A similar pattern of localization was seen for the *T. cruzi* homolog of AEK1 (Chiurillo et al., 2021). The preliminary differential digitonin permeabilization studies indicated that AEK1 is not present within organelles, and it is not tightly associated with the cytoskeleton. Thus, the nature of the AEK1 puncta remains unresolved.

## 4 Discussion

In our previous work, we validated AEK1 as a drug target in *T. brucei*. Here, we add evidence in support of developing inhibitors of AEK1 by showing that less than 5% of the live (motile) cells remaining after inhibition of AEK1 for 24 h are able to give rise to a clonal population *in vitro*. Even after just 8 h, when no visible defect was detected, a significant proportion of cells are unable to clonally expand. This is in contrast to certain other targets where inhibition leads to arrest of cell growth but growth can resume once the inhibitor is removed (Worthen et al., 2010). The relatively rapid loss of viability of cells on inhibition of AEK1 activity suggests that any drug developed to target AEK1 would need only a relatively short treatment regime. These data encouraged us to screen a small kinase-directed library for compounds that could bind the AEK1 ATP-binding site. The Lantha-TR-Fret assay that we developed for screening compounds that bind AEK1 does not require the protein to be active, removing the requirement for phosphorylation of serine 229 in the activation loop. It is also sensitive enough that it works with relatively low levels of protein. In the screen, we identified 17 compounds that reduced the signal by 50% or more. This relatively low hit rate suggests that AEK1 may be difficult to inhibit with currently available inhibitors. The most active compound in the screen, hesperadin, has been previously described as an inhibitor of the *T. brucei* homolog Aurora kinase, AUK1 (Jetton et al., 2009). The identification of hesperadin provided us with a useful tool to assess the integrity of the ATP-binding site of mutant AEK1 proteins. Hesperadin’s ability to inhibit two distinct PKs in *T. brucei* leads to the intriguing possibility of targeting two separate and essential molecules with one drug. In the bloodstream forms, hesperadin treatment (Jetton et al., 2009) and genetic depletion of AUK1 (via RNAi) or AEK1 (via conditional knockouts) all yield the same phenotype, at least at the level of the current analyses. One possibility is that AUK1 and AEK1 are epistatic (with one directly or indirectly activating the other), with both being inhibited by hesperadin.

Our data show that, as with most AGC kinases, the HM of AEK1 is essential for functionality. Mutation of the hydrophobic phenylalanine residues to alanine completely abrogated the function of the protein in the genetic rescue studies. Furthermore, mutation of the adjacent (phosphorylated) serine residue led to a slow growth phenotype, demonstrating the importance of this residue, as is seen in many other AGC kinases. For AKT1, the HM serves a dual function, the first being recruitment of PDK1 leading to phosphorylation of the AKT1 activation loop, and the second being a *cis* interaction with its own PIF pocket. However, AKT1 can also be activated via an HM-independent mechanism via a PH–domain interaction with second messenger PtdIns(3,4,5)P3 (Najafov et al., 2012), an avenue not available to AEK1. The S395A data are consistent with an essential *cis* or *trans* interaction (or both) for the role of the HM. The requirement for an intact PIF pocket points to an essential *cis* interaction.

Autophosphorylation by AEK1(S395A) immunopurified from *T. brucei* was relatively intact, but the protein showed almost no transphosphorylation of an exogenous (non-physiological) substrate *in vitro.* Given that AEK1(S395A) partially rescued function *in vivo*, and that phosphorylation of distinct substrates is likely to be the most relevant function of AEK1, this finding is somewhat perplexing. The expression levels of AEK1(S395A) relative to other clones suggests that differential overexpression does not explain the ability to rescue. However, compared with endogenous AEK1, these proteins are probably overexpressed in *T. brucei* due to the β-tubulin and actin untranslated regions (UTRs) used to express the mutant proteins; this may facilitate rescue via a suboptimal enzyme. A more trivial possibility is that the S395A defect may be more pronounced *in vitro* because the substrate used in the assay was suboptimal. Another alternative is that molecular interactions may bolster AEK1 activity *in vivo*, and that these interactions are lost in the immunoprecipitation process. For example, the puncta bearing AEK1 (seen in immunofluorescence microscopy) may provide a hub that facilitates access to substrates or regulatory proteins.

Our findings indicate that the AGC kinase AEK1 is controlled by a set of structural interactions and phosphoregulatory events that parallel those seen in several human AGC kinases and have been conserved in the deeply branched eukaryote *T. brucei*. All of the hallmark phosphorylation sites are present in AEK1. When measured by functional studies, the ability of the alanine replacements at these sites run the gamut from total failure (S229A) to partial rescue (T376A and S395A) and, finally, apparently complete rescue (S221A). The inability of AEK1(S229A) to rescue the conditional knockout provides strong evidence of an essential role for phosphorylation of this residue. While phosphomimetics often can functionally replace phosphoresidues, we observed that aspartic acid could not replace serine at this site. This is not entirely unexpected since phosphomimetic substitutions in the activation loops of several other PKs, including AGC kinases, showed highly decreased activity in cellular assays. For example, when the corresponding phosphothreonine residue in AKT1 was replaced by the phosphomimetic aspartic acid, diminished oncogenic transformation was observed (Najafov et al., 2012). Even phosphoserine only partially rescued phosphothreonine in AKT1 (Balasuriya et al., 2018). The phosphomimetic replacements in the activation loop did not rescue AKT3 (Brodbeck et al., 1999) and showed only partial rescue in CDK9 (Ramakrishnan and Rice, 2012). At the structural level, a phosphomimetic replacement at the RSK activation loop can interact with only two of the three critical basic residues (Somale et al., 2020), and a phosphomimetic at the activation loop of PAK1 cannot interact with a critical lysine in the alpha C helix and does not rescue function (Ng et al., 2010).

Considerable data exist concerning the activation of mammalian AGC kinases by phosphorylation. Most well studied is the phosphorylation of the activation loop of multiple AGC kinases by PDK1 (phosphoinositide dependent kinase 1, an AGC kinase itself). *T. brucei* contains a protein annotated as PDK1, Tb927.9.4910, based on the similarity of the kinase domain to the mammalian PDK1 and the presence of a putative phosphoinositide-binding domain. However, the putative phosphoinositide-binding domains of AEK1 (FYVE-4) and human PDK1(PH) are not related (Stahelin et al., 2014). Although the kinase domains of human PDK1 and Tb927.9.4910 show a high degree of similarity, the kinase domain of the human PDK1 is most similar to the *T. brucei* protein kinase A. Moreover, the phenotype of parasites depleted for Tb927.9.4910 by RNAi does not cause cell death (Alsford et al., 2011), as would be expected if that protein were responsible for activation of AEK1 and other AGC kinases, showing instead a slow growth phenotype (Jones et al., 2014). However, it should be noted that these experiments did not verify the extent of the RNAi knockdown. We suggest that the question of whether or not Tb927.9.4910 encodes a functional homolog of the mammalian PDK1 should be revisited.

## Data availability statement

The original contributions presented in the study are included in the article/**Supplementary Material**. Further inquiries can be directed to the corresponding author.

## Author contributions

BJ: Conceptualization, Formal analysis, Funding acquisition, Investigation, Methodology, Project administration, Resources, Supervision, Writing – original draft, Writing – review & editing. MP: Conceptualization, Formal analysis, Funding acquisition, Project administration, Supervision, Writing – original draft, Writing – review & editing. BP: Investigation, Writing – review & editing. MD: Investigation, Writing – review & editing. AD: Investigation, Writing – original draft, Writing – review & editing. ZI: Investigation, Writing – review & editing. DM: Conceptualization, Funding acquisition, Supervision, Writing – review & editing.
