# Supplementary material for: An essential Trypanosoma brucei protein kinase: a functional analysis of regulation and the identification of inhibitors
Source: Front Parasitol. 2023 Nov 14;2:1272378. doi: 10.3389/fpara.2023.1272378 (PMC10720658; doi:10.3389/fpara.2023.1272378)
Supplement: Supplementary file 1 [file Image_1.pdf]

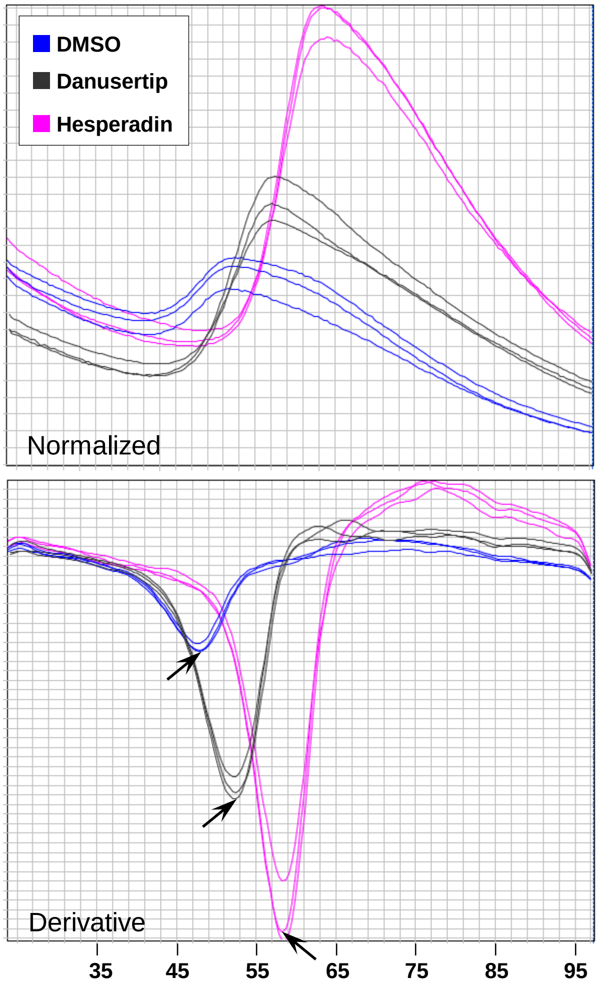


**Supplementary Figure 1**. Example of protein thermal shift for compounds binding wild-type AEK1 protein purified from *E. coli*. The top panel is the fluorescent signal for the protein as temperature is increase. The bottom panel is the first derivative of the curve. Arrows in the derivative curve denote the $T_m$ of the protein.

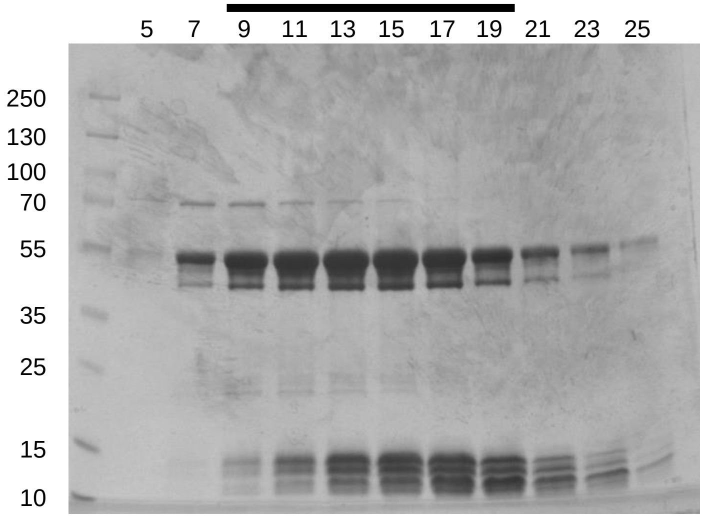


His-purification

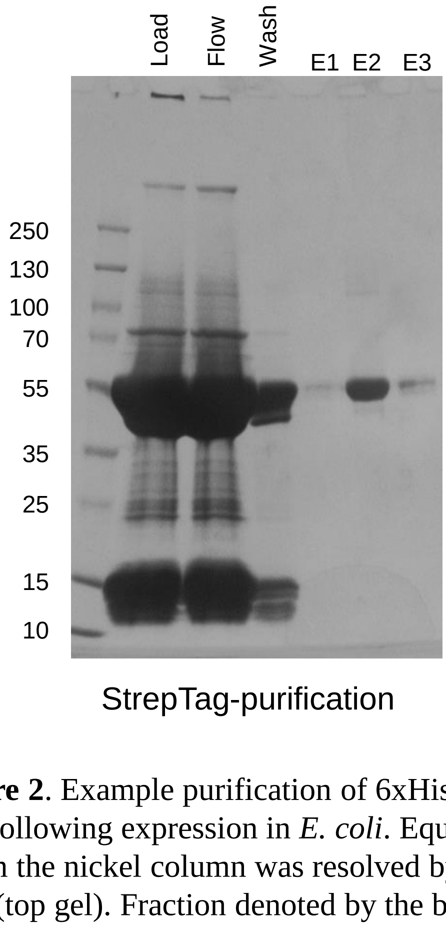


StrepTag-purification

**Supplementary Figure 2**. Example purification of 6xHis-AEK1(FF>AA)-Strep tagged protein  following expression in *E. coli*. Equal volumes from the indicated fraction from the nickel column was resolved by SDS-PAGE and stain with Coomassie (top gel). Fraction denoted by the black bar were pooled concentrated and the proteins repurified on a Streptactin column. Equal percentage of either the column load, flowthrough, wash and elute fractions were analyzed by SDS-PAGE (bottom gel).

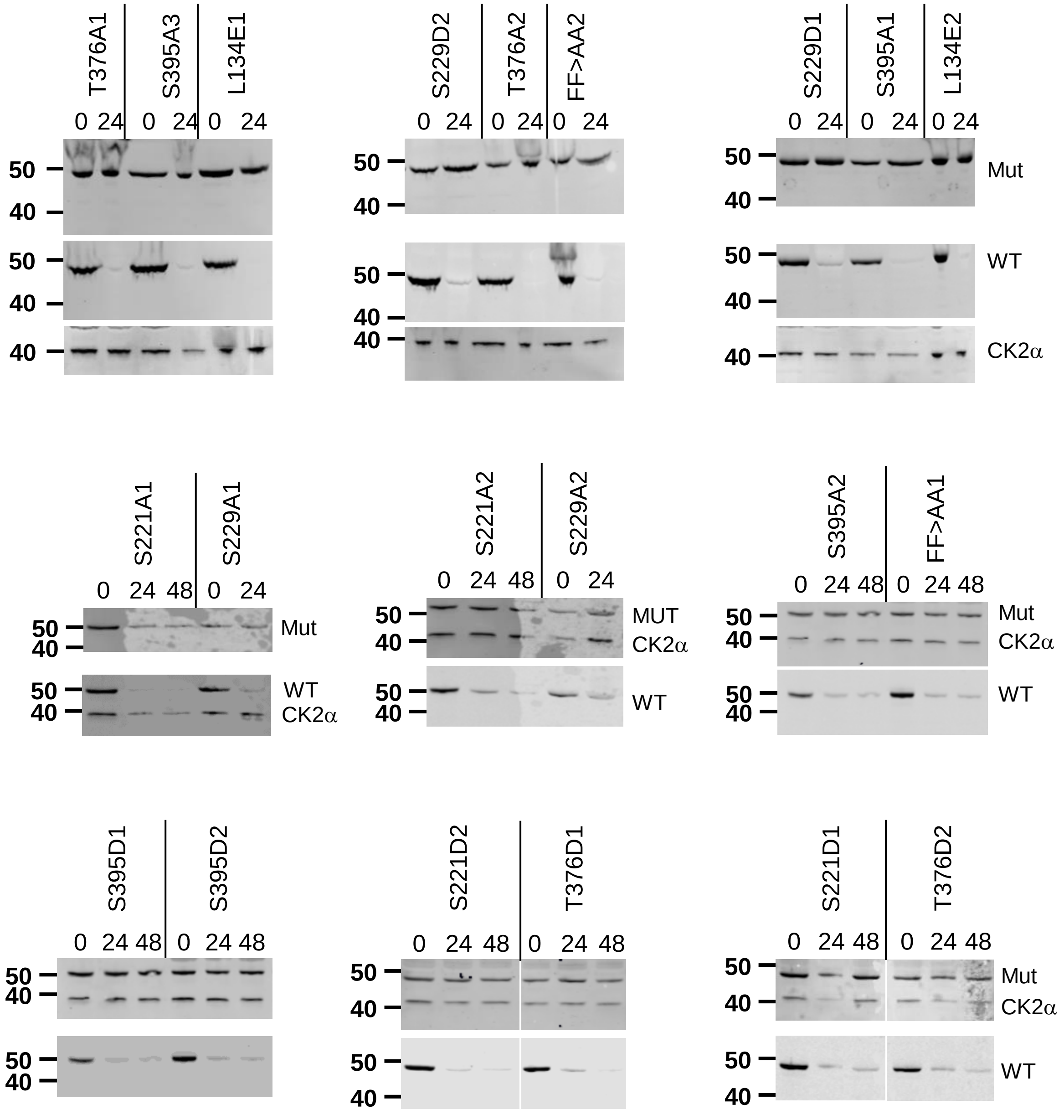


**Supplementary Figure 3**. Expression of epitope-tagged WT and mutant AEK1 proteins following Tet withdrawal. Samples were collected at the indicated number of hours following withdrawal from Tet. Blots were probed for HA (mutant AEK1 as indicated), V5 (Tet regulated WT protein) or the loading control CK2α. Samples shown in a group were all on the same gel. Migration of the molecular weight markers are shown on the left for each gel. Note WT AEK1 protein was highly reduced upon withdrawal of Tet.

.

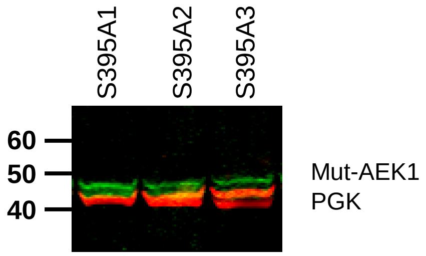


**Supplementary Figure 4**. Western blot comparing AEK1(S395A) expression in different clonal lines. AEK1 protein in green. PGK (red) was used as a loading control. The more severe phenotype of the S395A1 line compared to the other S395A lines is not explained by a lower level of expression.
