# Supplementary material for: An essential Trypanosoma brucei protein kinase: a functional analysis of regulation and the identification of inhibitors
Source: Front Parasitol. 2023 Nov 14;2:1272378. doi: 10.3389/fpara.2023.1272378 (PMC10720658; doi:10.3389/fpara.2023.1272378)
Supplement: Supplementary file 2 [file Table_1.docx]

Supplementary Table Primers

| Primer number | Primer Name | Sequence 5’>3’ |
| --- | --- | --- |
| 1 | AEK1-BglII-ATG | CCGAGATCTGATGCTCAATAGACTATTTGGCCG |
| 2 | AEK1-Stop-HindIII | CCGAAGCTTCTAACTACCACCACCCAGATGATTA |
| 3 | AEK1-Strep-S | TAATCATCTGGGTGGTGGTAGTGCAGGATGGAGCCACCCGCAG |
| 4 | AEK1-Strep-AS | CTGCGGGTGGCTCCATCCTGCACTACCACCACCCAGATGATTA |
| 5 | AEK1-pJX-ATG | TTTTGTTTAACTTTAAGAAGGAGATATACCATGCTCAATAGACTATTTGGCCG |
| 6 | AEK1-pJX-P1218M | GTGGTGGTGGTGGTGCTCGAGACTACCACCACCCAGATGATTA |
| 7 | pJX- reverse | GGTATATCTCCTTCTTAAAGTTAAACAAAATTATTTCTAGAG |
| 8 | pJK- forward | CTCGAGCACCACCACCACCAC |
| 9 | AEK1-L134E-S | CAGTCCGAGCA*T*AA*G***GAG**TTCTTTGTGATGGA |
| 10 | AEK1-L134E-AS | CTCCATCACAAAGAA**CTC***C*TT*A*TGCTCGGACTG |
| 11 | AEK1-S221A-S | CAAAATGGGTGAGAATGCA**G**CGGTAACGGACATGCG*C*GCGAACTCATTTGTTGGTTCACCATTTTATG |
| 12 | AEK1-S221A-AS | CATAAAATGGTGAACCAACAAATGAGTTCGC*G*CGCATGTCCGTTACCG**C**TGCATTCTCACCCATTTTG |
| 13 | AEK1-S221D-S | CAAAATGGGTGAGAATGCAG**AC**GTAACGGACATGCG*C*GCGAACTCATTTGTTGGTTCACCATTTTATG |
| 14 | AEK1-S221D-AS | CATAAAATGGTGAACCAACAAATGAGTTCGC*G*CGCATGTCCGTTAC**GT**CTGCATTCTCACCCATTTTG |
| 15 | AEK1-S229A-S | CAAAATGGGTGAGAATGCATCGGTAACGGACATGCG*C*GCGAAC**G**CATTTGTTGGTTCACCATTTTATG |
| 16 | AEK1-S229A-AS | GGTGAACCAACAAATG**C**GTTCGC*G*CGCATGTCCGTTACCGATGCATTCTCACCCATTTTG |
| 17 | AEK1-S229D-S | GTGAGAATGCATCGGTAACGGACATGCG*C*GCGAAC**G**ACTTTGTTGGTTCACCATTTTATG |
| 18 | AEK1-S229D-AS | CATAAAATGGTGAACCAACAAAGT**C**GTTCGC*G*CGCATGTCCGTTACCGATGCATTCTCAC |
| 19 | AEK1-T376A-S | AGTGGAAACAACTCATCAAAGAATCCCGCACAGGTTGT*C*AAC**G**CACCAGCACATTCGAGT |
| 20 | AEK1-T376A-AS | ACTCGAATGTGCTGGTG**C**GTT*G*ACAACCTGTGCGGGATTCTTTGATGAGTTGTTTCCACT |
| 21 | AEK1-T376D-S | AGTGGAAACAACTCATCAAAGAATCCCGCACAGGTTGT*C*AAC**GAC**CCAGCACATTCGAGT |
| 22 | AEK1-T376D-AS | ACTCGAATGTGCTGG**GTC**GTT*G*ACAACCTGTGCGGGATTCTTTGATGAGTTGTTTCCACT |
| 23 | AEK1-F391A ,F394A-S | CTCGGCAGCAGCAACTC**GC**TAACGGG**GC**TTCATGTAC*A*ACTGATAATCATCTGGGTG |
| 24 | AEK1-F391A, F394A-AS | CACCCAGATGATTATCAGT*T*GTACATGAA**GC**CCCGTTA**GC**GAGTTGCTGCTGCCGAG |
| 25 | AEK1-S395A-S | CTCGGCAGCAGCAACTCTTTAACGGGTTT**G**CATGTAC*A*ACTGATAATCATCTGGGTG |
| 26 | AEK1-S395A-AS | CACCCAGATGATTATCAGT*T*GTACAT**GC**AAACCCGTTAAAGAGTTGCTGCTGCCGAG |
| 27 | AEK1-S395D-S | CTCGGCAGCAGCAACTCTTTAACGGGTTT**GAC**TGTAC*A*ACTGATAATCATCTGGGTG |
| 28 | AEK1-S395D-AS | CACCCAGATGATTATCAGT*T*GTACA**GTC**AAACCCGTTAAAGAGTTGCTGCTGCCGAG |

Underlined sequences are restriction sites used for cloning. Bases used to generate listed mutation are in bold and underlined while bases in italics were used to either disrupt secondary structure in the primer or either generate or disrupt a restriction site to facilitate identification of the linked mutation.
